# Comparing amniotic fluid mass spectrometry assays and amniocyte gene analyses for the prenatal diagnosis of methylmalonic aciduria

**DOI:** 10.1371/journal.pone.0265766

**Published:** 2022-03-31

**Authors:** Yupeng Liu, Zhehui Chen, Lulu Kang, Ruxuan He, Jinqing Song, Yi Liu, Chunyan Shi, Junya Chen, Hui Dong, Yao Zhang, Yanyan Ma, Tongfei Wu, Qiao Wang, Yuan Ding, Xiyuan Li, Dongxiao Li, Mengqiu Li, Ying Jin, Jiong Qin, Yanling Yang

**Affiliations:** 1 Department of Pediatrics, Peking University People’s Hospital, Beijing, China; 2 Department of Pediatrics, Peking University First Hospital, Beijing, China; 3 Department of Gynaecology and Obstetrics, Peking University First Hospital, Beijing, China; 4 Department of Pediatrics, Qinghai University Affiliated Hospital, Xining, China; 5 Clinical Laboratory Center, Capital Medical University, Beijing, China; 6 Department of Endocrinology, Genetics and Metabolism, Beijing Children’s Hospital, Capital Medical University, Beijing, China; 7 Department of Precise Medicine, General Hospital of Tianjin Medical University, Tianjin, China; 8 Children’s Hospital Affiliated to Zhengzhou University, Zhengzhou, China; Fisheries and Oceans Canada, CANADA

## Abstract

**Background:**

Methylmalonic aciduria (MMA), a rare inherited disorder, is the most common organic aciduria in China, and prenatal diagnosis has contributed to its prevention. However, the prenatal diagnosis of MMA using cultured amniocytes or chorionic villi to detect gene mutations is exclusively applicable to families with a definite genetic diagnosis. To evaluate the reliability of mass spectrometry assays for the prenatal diagnosis of MMA, we conducted a retrospective study of our 10 years’ experience.

**Materials and methods:**

This retrospective compare study reviewed the medical records for maternal and fetuses data for 287 mothers with a family history of MMA from June 2010 to December 2020. Methylmalonate and propionylcarnitine in cell-free amniotic fluid were measured using a stable isotope dilution method (GC/MS) and MS/MS-based method (LC/MS/MS). Total homocysteine (tHcy) was measured by fluorescence polarization immunoassay. Depending on the presence of disease-causing gene mutations in probands, gene studies on amniocytes from 222 pregnant women were performed.

**Results:**

For 222 fetuses of the families with definite genetic diagnosis, gene analyses were performed using cultured amniocytes. 52 fetuses were affected by MMA, whereas 170 were “unaffected”. For GC/MS and LC/MS/MS, the specificity was 96.5% and 95.9%, sensitivity was 71.2% and 84.6%, respectively. The positive and negative predictive values were 86.0% and 91.6% and 86.3% and 95.3%, respectively. Propionylcarnitine/butyrylcarnitine ratio showed the highest accuracy and could thus serve as a sensitive indicator to identify those at a risk for MMA. When GC/MS and LC/MS/MS were performed in parallel, the specificity was 92.5% and sensitivity was 95.6%. When evaluating tHcy, the positive and negative predictive values were 95.0% and 96.1%, respectively. In 65 fetuses without family genetic diagnosis, 11 were finally confirmed to have MMA and 54 were “unaffected” by amniotic fluid biochemical assays. The 54 children showed normal urine organic acids and healthy development after birth.

**Conclusions:**

Amniotic fluid biochemical assays using GC/MS and LC/MS/MS in parallel increased the accuracy of prenatal diagnosis of MMA. Propionylcarnitine is a more reliable marker than methylmalonic acid in amniotic fluid. Further, tHcy is recommended for the prenatal diagnosis of combined MMA and homocysteinemia.

## Introduction

Methylmalonic aciduria (MMA), a rare inherited disorder, is the most common organic aciduria in Mainland China. MMA comprise a group of genetically heterogeneous autosomal recessive disorders caused by defective metabolic pathways involving methylmalonyl-CoA mutase (MUT) or its cofactor, cobalamin [1, 2]. MMA encompass 15 diseases caused by the deficient MUT activity or other defects in adenosylcobalamin biosynthesis [3, 4]. The clinical manifestations of the patients are complex, ranging from asymptomatic to death. The disease could occur at any age, from prenatal to adult [5, 6]. MMA can be diagnosed using mass spectrometry and genetic approaches [4, 7]. With advancements in neonatal screening and selective screening, an ever-increasing number of Chinese patients are being diagnosed with clinical and genetic heterogeneous disorders. Prenatal diagnosis has substantially helped in improving the quality of life of patients and their families.

The golden-standard method of prenatal diagnosis of MMA is gene study of cultured amniocytes or chorionic villi [8, 9]. However, this method is exclusively applicable to the families with a definite genetic diagnosis. Mass spectrometry assays such as liquid chromatography–tandem mass spectrometry (LC/MS/MS) for acylcarnitine quantification and stable isotope dilution methods using gas chromatography–mass spectrometry (GC/MS) for methylmalonate determination in small amounts of amniotic fluid have been developed. These biochemical methods has the advantage of fast and accurate [10–12]. Herein we assessed families with a history of MMA with the aim of evaluating the accuracy as well as efficacy of mass spectrometry assays and gene analyses for the prenatal diagnosis of MMA.

## Materials and methods

### Subjects and diagnosis

From June 2010 to December 2020, we enrolled 287 unrelated pregnant women with a family history of MMA. The probands of the families were born at term after uneventful pregnancies and deliveries from healthy non-consanguineous parents. MMA diagnosis was based on a well-established criteria [7]. Blood amino acid and acylcarnitine profiles of the probands were analyzed by LC/MS/MS (Waters MS/MS system A, 1445–002, Milford, MA; API 3200, Applied Biosystems, Foster City, CA) and the ChemoView^™^ software. MMA was suspected if an increase in propionylcarnitine (C3) (> 5.0 μmol/L; normal range 1.0–5.0 μmol/L), propionylcarnitine/acetylcarnitine ratio (C3/C2 > 0.50, normal range 0.10–0.50), and propionylcarnitine/free carnitine ratio (C3/C0 > 0.25, normal range 0.03–0.25) was noted. Urinary organic acid profiles were analyzed using GC/MS (Shimadzu QP 2010, Shimadzu Corporation, Kyoto, Japan) and the Inborn Errors of Metabolism Screening System software for the differential diagnosis of organic acidurias. If urinary methylmalonic acid and its metabolites were continuously higher and the presence of secondary MMA due to cobalamin deficiency had been excluded, the diagnosis of inherited MMA was established. Serum total homocysteine (tHcy) were measured by fluorescence polarization immunoassay (normal range 4.0–12.0 μmol/L). When serum tHcy was higher than the normal control, the diagnosis of combined MMA and homocysteinemia was established; if not, isolated MMA was diagnosed [7, 13, 14].

Of 287 families, 65 probands (22.6%) died in the early infancy without genetic diagnosis. They were diagnosed by biochemical studies. 48 of them had combined MMA and homocysteinemia and 17 had isolated MMA. 222 probands got definite genetic diagnosis [4, 15]. Mutation analysis followed the recommendations of the Human Genome Variation Society (http://www.hgvs.org/mutnomen). Sequence data were compared with an integrated set of variants, genotypes, and haplotypes from the 1000 Genomes Project (www.1000genomes.org). 169 probands had combined MMA and homocysteinemia cblC type caused by MMACHC gene mutations. 53 probands had isolated MMA caused by MMUT gene mutations (Table 1).

**Table 1:** Biochemical and genetic diagnosis of 287 probands with methylmalonic aciduria.

Probands	Total		Without definite genetic diagnosis		With definite genetic diagnosis		
	N	%	N	%	Causative gene	N	%
Combined MMA and homocysteinemia	217	75.6	48	16.7	*MMACHC*	169	58.9
Isolated MMA	70	24.4	17	5.9	*MMUT*	53	18.5
Total	287	100.0	65	22.6		222	77.4

N = number, MMA = methylmalonic aciduria.

### Amniotic fluid sample collection

All 287 pregnant women provided written informed consents after the potential advantages and risks of amniocentesis were discussed with them. Amniotic fluid samples were obtained by amniocentesis at 16–22 weeks of gestation at the Department of Obstetrics and Gynecology. 15–20 mL of amniotic fluid was collected from each woman and centrifuged (3000 rpm, 5 min, 4°C). The cell-free amniotic fluid was then separated for biochemical assays, whereas amniocytes were cultured using standard methods for gene analyses [16, 17].

### Biochemical assays

Amino acid and acylcarnitine profiles of amniotic fluid were analyzed using LC/MS/MS (Waters MS/MS system A, 1445–002, Milford, MA; API 3200, Applied Biosystems, Foster City, CA) and the concentrations of the metabolites were calculated automatically with the ChemoView^™^ software [12, 18]. MMA was suspected in the fetuses if two or three of these markers (C3, C3/C0, C3/C2), were elevated [12, 18, 19].

Organic acid profiles of amniotic fluid were analyzed by stable isotope dilution methods using GC/MS (Shimadzu QP 2010, Shimadzu Corporation, Kyoto, Japan), according to a previous established protocol [9, 10, 20]. Mass spectra were obtained by standard electron impact ionization scanning from 50 to 500 m/z. Data were collected with gas chromatography/mass spectrometry solution software. If methylmalonic acid and its metabolites were elevated, the diagnosis of MMA was established [21]. tHcy in amniotic fluid was determined by fluorescence polarization immunoassay (Abbott I2000, Abbott Park, IL). If tHcy was also elevated, combined MMA and homocysteinemia in the fetus was diagnosed.

### Gene analyses

Depending on the genetic diagnosis of probands (Table 1), mutation analyses of amniocytes were performed.

Genomic DNA was extracted from cultured amniocytes using the Gen Midi Kit. The whole coding region sequence and exon–intron boundary sequence were amplified by PCR. Purified amplicons were then sequenced on an ABI 3730XL genetic analyzer, and mutation analyses were performed using DNASTAR^®^ (Madison, WI, USA). The analyzed sequences were compared with cDNA and genomic DNA sequences in GenBank. Sequences of proteins homologous to human *MMACHC* and *MMUT* were obtained using BLAST. Sequence data were compared with those obtained from probands, parents, and normal controls.

### Evaluation using MS-based methods

ROC curve analyses were performed to identify parsimonious subsets of the seven MMA-related markers including methionine (Met), free carnitine (C0), propionylcarnitine (C3), propionylcarnitine/free carnitine ratio (C3/C0), propionylcarnitine/acetylcarnitine ratio (C3/C2), propionylcarnitine/butyrylcarnitine ratio (C3/C4), and propionylcarnitine/palmitoylcarnitine ratio (C3/C16). The AUC ranged from 0 to 1 and indicated the ability of a test to discriminate which measure(s), whether independent or in combination, were better for assessing MMA.

For the purpose of primary analyses, we evaluated the performance of GC/MS and LC/MS/MS using sensitivity, specificity, and positive and negative predictive values relative to the gene finding. Gene approach was considered the standard against which the determination of methylmalonic acid and its metabolites by MS-based assays.

Sensitivity, specificity, and positive and negative predictive values were calculated using standard formulae for a binominal proportion, and 95% CIs were calculated by the Wilson interval method. Because our study was designed to show that MS-based assays are not inferior to the “gold standard” approach (gene analysis), we used a one-sided hypothesis, which was tested using the chi-square method to compare differences between the sensitivity and specificity of the two approaches, with a predetermined clinically meaningful limit of 0.05.

### Statistical analysis

Continuous data have been summarized using mean or median. Categorical data were analyzed using counts and percentages. The chi-square test was used for examining categorical data. The area under the receiver operator characteristic (ROC) curve (AUC) was calculated, and 95% confidence interval (CI) was used to test the hypothesis that AUC = 0.5. SPSS 26.0 was used for statistical analyses.

### Ethics statement

This study was approved by the Hospital Institutional Ethics Committee and was conducted in accordance with the Declaration of Helsinki. Written informed consent was obtained from the parents of all patients for sample collection and publication of medical data.

## Results

287 amniotic fluid samples were analyzed to determine the risk of MMA in the fetuses (Table 2).

**Table 2:** Prenatal diagnosis of 287 fetuses with a family history of methylmalonic aciduria.

Diagnosis of probands	Combined MMA and homocysteinemia				Isolated MMA				Control		Units
**By biochemical and genetic studies**	**Affected N = 43**		**Unaffected N = 126**		**Affected N = 9**		**Unaffected N = 44**		**N = 248**		
	**median**	**range**	**median**	**range**	**median**	**range**	**median**	**range**	**P 2.5**	**P 97.5**	
Metabolic markers											
Met	14.1	5.7–40.0	16.7	2.8–40.7	14.2	8.7–22.5	15.8	5.7–30.9	8.3	31.0	μmol/L
C0	14.5	5.7–26.5	17.0	7.6–41.3	14.1	6.3–21.4	17.3	8.3–41.4	7.7	27.1	μmol/L
C3	4.1	0.7–9.2	1.1	0.2–6.0	5.1	0.7–9.9	1.2	0.3–2.8	0.3	2.5	μmol/L
C3/C0	0.29	0.01–0.77	0.07	0.01–0.34	0.39	0.03–0.59	0.07	0.01–0.18	0.02	0.14	
C3/C2	0.45	0.10–1.13	0.13	0.03–0.42	0.71	0.08–1.45	0.13	0.04–0.37	0.04	0.26	
C3/C4	7.36	1.58–17.67	2.13	0.45–17.77	8.87	1.34–24.63	1.99	0.42–3.48	0.57	5.69	
C3/C16	90.56	5.80–419.56	22.57	0–81.55	198.06	8.74–624.66	27.99	2.81–96.38	0	82.20	
MMA	12.1	0–122.7	0.1	0–7.4	24.2	0–79.4	0.8	0–2.7	0	0.2	μmol/L
tHcy	14.7	2.13–24.9	2.4	1.0–14.7	1.8	1.2–3.9	1.7	1.0–4.3	4.0	12.0	μmol/L
Mutations											
Two mutations (N)	43		0		9		0				
One mutation (N)	0		88		0		24				
No mutation (N)	0		38		0		20				
**By biochemical assays**	**Affected N = 8**		**Unaffected N = 40**		**Affected N = 3**		**Unaffected N = 14**		**Contro N = 248**		
	**mean**	**range**	**mean**	**range**	**mean**	**range**	**mean**	**range**	**P 2.5**	**P 97.5**	
Metabolic markers											
Met	14.5	10.3–40.4	13.5	6.7–25.5	11.1	10.4–14.8	13.5	7.3–17.8	8.3	31.0	μmol/L
C0	13.2	8.7–20.0	13.6	6.5–34.7	12.9	12.7–19.5	14.0	10.2–17.1	7.7	27.1	μmol/L
C3	4.9	3.2–13.5	0.9	0.2–2.2	2.7	2.4–3.4	1.0	0.1–2.0	0.3	2.5	μmol/L
C3/C0	0.45	0.20–0.90	0.07	0.02–0.24	0.14	0.03–0.67	0.08	0.01–0.14	0.02	0.14	
C3/C2	0.60	0.50–2.23	0.10	0.05–2.47	0.29	0.06–0.54	0.15	0.01–0.14	0.04	0.26	
C3/C4	6.32	3.91–13.72	1.90	0.73–4.63	12.27	5.24–21.00	1.68	0.93–3.78	0.57	5.69	
C3/C16	90.97	54.19–264.23	15.87	0–49.00	20.08	0–8.97	8.11	4.87–49.00	0	82.20	
MMA	24.2	5.1–37.4	0	0	17.0	10.1–279.9	0	0–0.8	0	0.2	μmol/L
tHcy	19.1	14.4–22.8	2.8	1.3–4.2	2.1	1.2–3.5	2.02	1.4–4.3	4.00	12.0	μmol/L

Notes: N = number of cases; Met = methionine; C0 = free carnitine; C3 = propionylcarnitine; C3/C0 = propionylcarnitine/free carnitine ratio; C3/C2 = propionylcarnitine/acetylcarnitine ratio; C3/C4 = propionylcarnitine/butyrylcarnitine ratio; C3/C16 = propionylcarnitine/palmitoylcarnitine ratio; MMA = methylmalonic acid; tHcy = total homocysteine.

222 (77.4%) samples were simultaneously tested using mass spectrometry assays and gene analyses. MMA was confirmed in 52 (23.4%) fetuses, whereas 170 (76.6%) fetuses were identified to be “unaffected.” In the 169 families with a history of combined MMA and homocysteinemia, 43 (25.4%) and 126 (74.6%) fetuses were identified to be "affected" and "unaffected”, respectively. Further, in 53 families with a history of isolated MMA, 9 (17.0%) fetuses were “affected” and 44 (83.0%) were “unaffected.”

**Fig 1:**
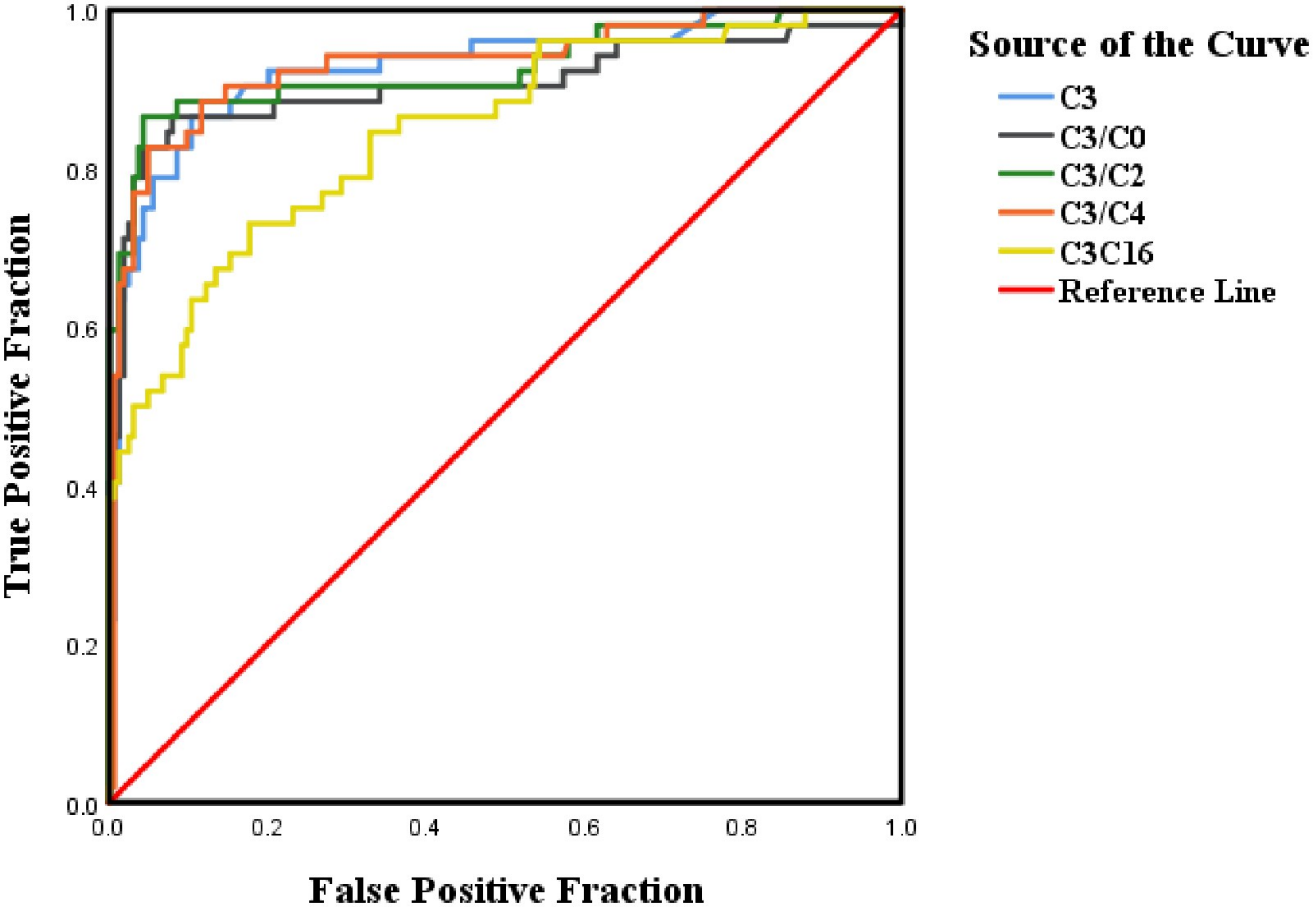
Receiver operating characteristic curves of C3, C3/C0, C3/C2, C3/C4, and C3/C16 for the diagnostic signature. True positive results (sensitivity) vs false positive results (1 − specificity) to identify the most reliable diagnostic signature. C3 = propionylcarnitine; C3/C0 = propionylcarnitine/free carnitine ratio; C3/C2 = propionylcarnitine/acetylcarnitine ratio; C3/C4 = propionylcarnitine/butyrylcarnitine ratio; C3/C16 = propionylcarnitine/palmitoylcarnitine ratio.

65 (22.6%) of the amniotic fluid samples were analyzed by biochemical assays because the probands of the families had not undergone gene diagnosis. 11 (16.9%) fetuses were affected by MMA. 8 (12.3%) of them were diagnosed as having combined MMA and homocysteinemia. 3 (4.6%) fetuses had isolated MMA. The remaining 54 fetuses were identified to be “unaffected” (Table 2). The AUC and 95% CIs of the seven MMA-related markers in 222 amniotic fluid samples which were confirmed by gene studies have been analyzed (Table 3). The AUC values of C3, C3/C0, C3/C2, C3/C4, and C3/C16 were > 0.5. The P values of Met, C0, C3, C3/C0, C3/C2, C3/C4, and C3/C16 were < 0.01. The results pertaining to markers with AUC > 0.5 and P > 0.01 were shown as Fig 1. LC/MS/MS indicated that C3/C4 had the highest accuracy for evaluating MMA (AUC, 0.933; 95% CI, 0.888–0.978; P < 0.001); thus, we considered C3/C4 to be a sensitive indicator for identifying those at a risk for MMA.

**Table 3:** Evaluation of methylmalonic aciduria-related markers in 222 amniotic fluid samples.

Test	Area	P	95% Confidence Interval	
			Lower bound	Upper bound
Met	0.351	< 0.01	0.263	0.439
C0	0.376	< 0.01	0.287	0.465
C3	0.929	< 0.01	0.884	0.975
C3/C0	0.907	< 0.01	0.845	0.969
C3/C2	0.928	< 0.01	0.876	0.979
C3/C4	0.933	< 0.01	0.888	0.978
C3/C16	0.848	< 0.01	0.784	0.911

Met = methionine; C0 = free carnitine; C3 = propionylcarnitine; C3/C0 = propionylcarnitine/free carnitine ratio; C3/C2 = propionylcarnitine/acetylcarnitine ratio; C3/C4 = propionylcarnitine/butyrylcarnitine ratio.

Increased methylmalonic acid in amniotic fluid analyzed by GC/MS indicated that 37 of the 222 fetuses had MMA, whereas 164 were “unaffected”. Six cases were false positives and 15 were false negatives comparing with gene studies (Table 4). Increased C3 analyzed by LC/MS/MS indicated that 44 fetuses were “affected” by MMA. 163 fetuses were “unaffected”. False positives results were observed in 7 samples. False negatives data were confirmed in 8 cases (Tables 4 and 5).

**Table 4:** Organic acids in 222 amniotic fluid samples tested by the stable isotope dilution method using GC/MS.

	Affected	Unaffected	False positives	False negatives
Number of fetuses	37 (16.7%)	164 (73.9%)	6 (2.7%)	15 (6.8%)
MMA (μmol/L)				
median	20.0	0.0	3.2	0.0
range	0.40–122.7		0.50–7.4	

MMA = methylmalonic acid.

**Table 5:** Methionine and acylcarnitines in 222 amniotic fluid samples tested using LC/MS/MS.

	Affected N = 44		Unaffected N = 163		False positives N = 7		False negatives N = 8	
	median	range	median	range	median	range	median	range
**Met (μmol/L)**	13.8	5.7–40.0	16.5	2.8–40.7	15.2	4.8–23.5	15.5	9.1–27.1
**C0 (μmol/L)**	13.6	5.7–26.5	16.9	7.6–41.3	20.0	9.8–41.4	18.8	10.1–26.5
**C3 (μmol/L)**	4.8	0.7–9.9	1.0	0.2–2.7	3.3	2.3–6.0	1.4	0.7–2.3
**C3/C0**	0.35	0.01–0.77	0.07	0.01–0.19	0.20	0.06–0.34	0.08	0.03–0.19
**C3/C2**	0.55	0.10–1.45	0.12	0.03–0.35	0.29	0.12–0.42	0.19	0.08–0.39
**C3/C4**	8.52	1.58–24.63	1.89	0.42–5.54	6.27	2.34–17.77	2.70	1.34–4.17
**C3/C16**	125.54	17.00–624.66	22.80	0–90.27	49.05	5.13–96.38	19.08	5.80–33.18

N = number of cases; Met = methionine; C0 = free carnitine; C3 = propionylcarnitine; C3/C0 = propionylcarnitine/free carnitine ratio; C3/C2 = propionylcarnitine/acetylcarnitine ratio; C3/C4 = propionylcarnitine/butyrylcarnitine ratio; C3/C16 = propionylcarnitine/palmitoylcarnitine ratio.

We then compared the data of biochemical markers determined by GC/MS and LC/MS/MS with the results of amniocyte gene analysis. The positive and negative predictive values for GC/MS were 86.0% and 91.6%, respectively, whereas those for LC/MS/MS were 86.3% and 95.3%, respectively. For GC/MS and LC/MS/MS, the specificity was 96.5% and 95.9%, sensitivity was 71.2% and 84.6%, and overall accuracy was 90.5% and 93.2%, respectively, compared with gene analysis.

Using paired chi-square test, we found no statistically significant differences in the specificity and sensitivity of GC/MS and LC/MS/MS (P > 0.05). When GC/MS and LC/MS/MS were performed in parallel, the specificity was 92.5% and the sensitivity was 95.6%.

We also compared the results of tHcy in amniotic fluid with amniocyte gene analysis. The positive and negative predictive values were 95.0% and 96.1%, respectively. The specificity, sensitivity, and accuracy were 98.4%, 88.4%, and 95.7%, respectively.

For the prenatal diagnosis of 65 fetuses from the families without genetic diagnosis, increased biochemical markers in 11 (16.9%) amniotic fluid samples checked by GC/MS and LC/MS/MS in parallel indicated the fetuses were affected by MMA (Table 6). 8 (12.3%) of them were diagnosed as having combined MMA and homocysteinemia. 3 (4.6%) fetuses had isolated MMA. The remaining 54 (83.1%) fetuses were considered to be unaffected by MMA (Table 2).

**Table 6:** Methylmalonic aciduria-related markers in amniotic fluid samples detected using GC/MS and LC/MS/MS.

	Affected (N = 11)		Unaffected (N = 54)	
	median	range	median	range
**MMA (μmol/L)**	23.4	5.1–279.9	0	0–0.8
**Met (μmol/L)**	14.0	10.3–40.4	13.5	6.7–25.5
**C0 (μmol/L)**	13.1	8.7–20.0	13.6	6.5–34.7
**C3 (μmol/L)**	3.9	2.4–13.5	0.9	0.1–2.2
**C3/C0**	0.41	0.03–0.90	0.07	0.01–0.24
**C3/C2**	0.54	0.06–2.23	0.10	0.01–2.47
**C3/C4**	6.83	3.91–21.00	1.79	0.73–4.63
**C3/C16**	82.65	0–264.23	17.21	0–54.87

N = number of cases; MMA = methylmalonic acid; Met = methionine; C0 = free carnitine; C3 = propionylcarnitine; C3/C0 = propionylcarnitine/free carnitine ratio; C3/C2 = propionylcarnitine/acetylcarnitine ratio; C3/C4 = propionylcarnitine/butyrylcarnitine ratio; C3/C16 = propionylcarnitine/palmitoylcarnitine ratio.

### Follow-up

224 fetuses without MMA were born. 204 children are now 2 to 12 years old with normal development and metabolic findings. Two children died in the accident. Three fetuses with combined MMA and homocysteinemia were born. Their mothers took mecobalamine (1 mg/day) and L-carnitine during pregnancy. Three patients were born at term. Abnormal blood and urinary metabolic profiles validated the prenatal diagnosis of combined MMA and homocysteinemia. Two patients began regular metabolic treatment immediately after birth. They are aged 3 and 12 years old respectively, with normal psychomotor and physical development. Unfortunately, the treatment of one patient had been initialed after the onset of epilepsy at the age of 3 months because the parents did not understand the importance of early treatment. She is now 5 years old with delayed psychomotor development.

## Discussion

MMA is the most common symptomatic organic aciduria in China [7, 22]. From June 1998 to December 2020, 1266 MMA patients was diagnosed at our hospital [22, 23]. Several analytical methods have been developed for the prenatal diagnosis of MMA. The measurement of enzyme activity in cultured amniocytes is time consuming and difficult; moreover, contamination with maternal cells may result in the generation of false negative results [9, 24]. Gene analyses using amniocytes or chorionic villi require proband-related information [25]. Two MS-based methods for the prenatal diagnosis of MMA has been reported. Some organic acids in amniotic fluid can be analyzed by a stable isotope dilution method using GC/MS. Some amino acids and acylcarnitines analysis using LC/MS/MS is also helpful [9, 11, 26]. These methods can provide reliable results quickly and require only a small quantity of amniotic fluid.

Morel et al. performed prenatal diagnosis for MMA and inborn errors of cobalamin metabolism and transport. They assessed 117 high-risk pregnancies and identified 21 (18%) affected fetuses by amniotic fluid metabolite measurement using GC/MS or LCMS/MS and direct mutation analysis [25]. Further, Hasegawa and Zhang Y et al. performed prenatal diagnosis of MMA using two MS-based methods and/or amniotic fluid homocysteine assay, successively [9, 21]. Hasegawa et al. suggested that acylcarnitines degraded at room temperature in a week, but organic acids such as methylmalonate and methylcitrate were stable for at least 2 weeks. Such findings suggest that prenatal diagnosis using two or more methods in parallel is much better. Cavicchi et al. reported a concurrent genetic and biochemical method (GC/MS and LC/MS/MS) for prenatal diagnosis, which was performed at 11 weeks of gestation in a family with a proband affected by mut deficiency and homozygotes for the MMUT gene c.643G>A mutation [8]. They suggested that the most accurate method for prenatal diagnosis in the first trimester is genetic analyses, but data pertaining to mutations in the proband must be available. Inoue reported that using cell-free amniotic fluid samples, which had been dried on a filter paper and transported at ambient temperatures, generated reproducible and accurate results, implying that this method could be applied for clinical analysis [20]. These years, many studies for the prenatal diagnosis of MMA using two MS-based methods and gene analyses were reported [27–29]. The results suggested that mass spectrometry approaches are convenient method for improving the prenatal diagnosis of MMA, especially in the cases where the genetic results are inconclusive [29, 30]. The characteristic metabolites C3, C3/C2, methylmalonic acid, and methylcitrate in amniotic fluid were reliable biochemical markers for the prenatal diagnosis of MMA [29, 30]. Ting Chen et al. valued the Hcy characteristic metabolite appears to be a sensitive biomarker for the prenatal diagnosis of cblC defect [31]. However, no previous studies have as yet compared mass spectrometry assays and gene analyses to evaluate the reliability of MS-based methods for the prenatal diagnosis of MMA.

We designed the present study to assess whether biochemical assays are as efficient as traditional gene analyses and to determine which of the two approaches is more reliable for the prenatal diagnosis of MMA. We found that biochemical assays of amniotic fluid using LC/MS/MS was as reliable as gene analyses. The MS/MS-based method was significantly more accurate than the stable isotope dilution method; however, there were no statistically significant differences in specificity and sensitivity between these methods. Our findings support the use of MS/MS for evaluating high-risk pregnancies of MMA. And the results also indicate a benefit of amniotic fluid analysis using LC/MS/MS should be a standard biochemical test. Furthermore, our results of LC/MS/MS revealed that C3/C4 had the highest accuracy; thus, it could serve as a sensitive indicator when using amniotic fluid samples to identify those at a risk for MMA. If the proband had isolated MMA without genetic diagnosis, we recommend that two MS-based methods should be performed in parallel for amniotic fluid analyses. If the proband was diagnosed with combined MMA and homocysteinemia, tHcy in amniotic fluid should also be measured, which is a convenient and reliable method for the prenatal diagnosis of homocysteinemia [21].

Although amniotic fluid propionylcarnitine determination by MS/MS is more reliable for the prenatal diagnosis of MMA, it is important to note that the sensitivity and specificity of MS/MS is not 100%. The MS/MS method failed to identify 8 “affected” cases in this study, whereas the stable isotope dilution method did not identify 15 “affected” cases. The failure of the MS/MS method to identify 100% “affected” cases could be attributed to the instability of acylcarnitines in amniotic fluid samples on the filter paper conserved at room temperature [9]. Alternatively, free carnitine could have been lower, thereby influencing C3 and butyrylcarnitine levels. Further, we could not deduce the reasons why the stable isotope dilution method failed to identify 100% “affected” cases. We thus propose that using MS/MS and a stable isotope dilution method in parallel is optimal for the prenatal diagnosis of MMA. Moreover, we found that assessing tHcy levels by fluorescence polarization immunoassay in amniotic fluid samples is an accurate way to identify fetuses with combined MMA and homocysteinemia.

To conclude, we propose that amniotic fluid biochemical assays and amniocyte gene analyses should be used as complementary approaches. Further, if the pathogenic mutations is unknown, two MS-based methods should be performed in parallel to obtain reliable results. Biochemical analysis of amniotic fluid using GC/MS and/or LC/MS/MS appears to be a fast and accurate approach for the prenatal diagnosis of MMA, although including gene analyses in the process of diagnosis is highly recommended.

## Supporting information

S1 File TREND statement checklist. (PDF)
